# Demography of Total Joint Replacement Surgeries Performed in a Tertiary Care Hospital: A Cross-sectional Survey

**DOI:** 10.31729/jnma.6949

**Published:** 2021-11-30

**Authors:** Shrawan Kumar Thapa, Manoj Kandel, Sunil Panta, Bishwa Raj Adhikari

**Affiliations:** 1 Department of Orthopedics, Bharatpur Hospital, Chitwan, Nepal

**Keywords:** *demography*, *osteoarthritis*, *total hip replacement*, *total knee replacement*

## Abstract

**Introduction:**

Total joint replacement of hip and knee is considered as one of the most successful orthopedic surgeries in the twenty-first century because of the only solution to end-stage arthritis of these joints. The real burden of the problem is yet to be established in developing countries like Nepal. This study aims to describe the demographic findings of the joint replacement surgeries among total lower limb surgeries in a tertiary care hospital.

**Methods:**

This cross-sectional survey was conducted using the hospital records of 73 total joint replacement surgeries of the lower limb in the Department of Orthopedics of a tertiary care hospital from November 2016 to November 2020. Ethical approval was taken from the Institutional Review Committee (reference number: 077/78-011). Convenience sampling was done. Data analysis was done using the Statistical Package for the Social Sciences version 20. Point estimate at 95% Confidence Interval was calculated along with frequency and percentage for binary data.

**Results:**

There were 73 total joint replacement of hips and knees. Of which, 32 (43.84%) total hip replacements were done in which one (3.13%) patient had a simultaneous bilateral hip replacement in single-stage and the other one (3.13%) had two-stage bilateral hip replacement. Forty one (56.16%) total knee replacements were done in which 18 (24.65%) had a simultaneous bilateral knee replacement and five (6.85%) had a unilateral knee replacement.

**Conclusions:**

Total joint replacements of the hip were more common among the lower limb surgeries.

## INTRODUCTION

Total hip replacement (THR) and total knee replacement (TKR) are usually performed as curative treatment in end-stage arthritis. They are becoming increasingly prevalent and an increasing range of techniques and materials are now available aiming to relieve pain and restore joint function.^1-3^

Radiographic severity and patient-related pain and functions play a major role in surgeons' recommendation for total joint replacement.^4^ The most frequent condition for both THR and TKR is Osteoarthritis followed by Rheumatoid arthritis, Avascular necrosis, and fractures. Results are better with these procedures. The prevalence and incidence rates of THR and TKR depend on socioeconomic status and healthcare systems, patient preferences, and prevalence of osteoarthritis.^5^ Very few hospitals outside the capital of Nepal are performing total joint replacement surgery in our part of the world and there are few relevant studies on the topic.

This study aims to describe the demographic profile of patients undergoing lower limb total joint replacement surgeries in a tertiary care hospital.

## METHODS

This cross-sectional survey was conducted at the Orthopedic Department of Bharatpur Hospital over 4 years, from November 2016 to November 2020. The ethical clearance was taken from the Institutional Review Committee (IRC) of Bharatpur Hospital (Ref: 077/78-011) dated July 14, 2021. All the hospital records of patients above 18 years of age who underwent lower limb surgeries were included. Patients with incomplete records, missing data, revision replacement surgeries were excluded. Preoperative written consent was taken from all the patients. Convenience sampling was done and the sample size was calculated as,

n = Z^2^ × p × q / e^2^

  = (1.96)^2^ × (0.5) × (1-0.5) / (0.1)^2^

  = 68

Where,

n= required sample size,
Z= 1.96 at 95% Confidence Interval (CI),
p= prevalence taken as 50% for maximum sample size,
q= 1-p
e= margin of error, 10%

The minimum required sample size was 68. However, data was collected from 73 patient records. The variables collected were age, gender, address, preoperative diagnosis, type and side of arthroplasty. Complications after surgery were also recorded.

The postero-lateral approach was used for THR in lateral decubitus position and the midline anterior medial parapatellar approach was used for TKR.^6^ We used one gram of intravenous tranexamic acid thirty minutes prior to incision for both THR and TKR patients and used a tourniquet for TKR to reduce perioperative bleeding.^7^ All of our patients of hip and knee replacement were done under epidural anaesthesia followed by subcutaneous injection of low molecular weight heparin 40mg for 5 days from the first postoperative day to prevent deep venous thrombosis.^8^ Postoperative follow up of patients were done at1 month, 6 months and then yearly. All patients underwent cemented TKR, elderly osteoporotic patients underwent cemented THR and younger individuals underwent uncemented THR for easier later revision.^9^

Statistical analyses were performed using the Statistical Package for the Social Sciences software (version 20). Point estimate at 95% CI was calculated along with frequency and percentage for binary data.

## RESULTS

**Figure 1:**
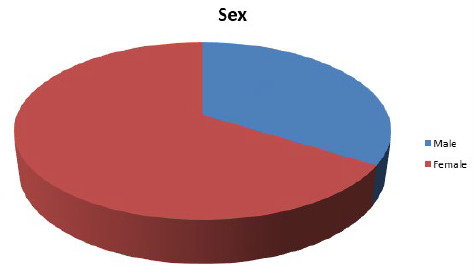
Gender-wise distribution of participants.

There were 53 patients among which 18 (34%) were male and 35 (66%) were female with a male to female ratio of 1:1.94 (Figure 1).

Age ranged from 21 years to 86 years with the mean age 56.91±15.12 years. The commonest cause of total hip and knee replacement was primary osteoarthritis in 27 (50.9%). In the hip, the second commonest cause was avascular necrosis of the femoral head 11 (20.8%) whereas in the knee the second commonest cause was rheumatoid arthritis 8 (15.1%) (Table 1).

**Table 1:** Preoperative diagnosis of study participants (n=53).

Diagnosis	Frequency
Primary Osteoarthritis	27 (50.9)
Rheumatoid arthritis	8 (15.1)
Tubercular arthritis	3 (5.7)
AVN	11 (20.8)
Other Inflammatory arthritis	4 (7.5)
Total	53 (100)

Others were fromMakwanpur, Kathmandu, Syangja, and Rukum. AmongTHR majority were left-sided 17 (56.67%) (Table 2).

**Table 2:** Side of THR and TKR.

Surgery	Side	Frequency n (%)
THR (n=30)	Right	11 (36.67)
	Left	17 (56.67)
	Bilateral	2 (6.66)
TKR (n=23)	Right	4 (17.39)
	Left	1 (4.35)
	Bilateral	18 (78.26)

Among TKR majority were bilateral 28 (78.26%) and all cemented (Table 3).

**Table 3:** Type of THR.

THR	Frequency (n = 32)
Cemented	4 (12.5)
Uncemented	28 (87.5)

Few complications encountered among which one (1.9%) TKR had a superficial infection that healed with antibiotics and secondary suture, another TKR in the valgus knee, one (1.9%), had common peroneal nerve (CPN) palsy that was recovered within three months of physiotherapy, two (3.8%) THR had perioperative periprosthetic fracture managed with circlage wiring and delayed weight-bearing, one (1.9%) THR had dislocation managed with closed reduction and traction for four weeks and three (5.7%) deaths within one and a half to two years (Table 4).

**Table 4:** Complications of THR and TKR.

Complications	n (%)
Periprosthetic fracture	2 (3.8)
Superficial wound infection	1 (1.9)
Dislocation	1 (1.9)
CPN palsy	1 (1.9)
Death	3 (5.7)
None	45 (84.9)

## DISCUSSION

Hip and knee replacement are common surgical procedures for better mobility and quality of life. The results showed similar demographic characteristics to those published studies,^5,10-12^ that is, the majority of patients were above 65 years and the commonest cause of total joint replacement was primary osteoarthritis followed by rheumatoid arthritis, avascular necrosis, and others.

Regarding gender, females have more incidence and prevalence of total knee replacement and males have more incidence and prevalence of total hip replacement as supported by various previous literature.^13,14^ Our patients had more female patients in TKR candidates whereas near an equal number of both genders in THR candidates. We had very few total joint replacement candidates compared to the developed world that might be because of lack of awareness, increased cost of implants, and no insurance policy.^15^ Most of our patients with TKR were simultaneous bilateral replacements which we found to be safe, effective with early functional recovery, higher patient satisfaction, and cost-effective in properly selected candidates similar to K.C KM et al.^16^ On geographical variation, most of our patients were local from Chitwan followed by nearby districts Nawalparasi, Makwanpur and some of them were far from Chitwan like Syanja, Rukum and Kathmandu.

Epidural anaesthesia has shown decreased incidence of deep venous thrombosis in these patients.^8^ All of our patients of hip and knee replacement were done under epidural anaesthesia followed by subcutaneous injection of low molecular weight heparin 40mg for 5 days from the first postoperative day to prevent deep venous thrombosis.

Total joint arthroplasty is associated with high blood loss that may need blood transfusions. The published studies favor the use of tranexamic acid as a safe and effective method of reducing blood loss.^7^ Our patients have no previous history of thromboembolic episodes and we used one gram of intravenous tranexamic acid thirty minutes before incision to reduce perioperative bleeding.

Although THR and TKR are relatively safe procedures, complications related to or not related to surgery were observed, warning of necessary precautions. Surgical site infection is a serious adverse event in total joint replacement. The incidence of infection in TKR is around 2%. We encountered one superficial wound infection in a lady with total knee replaced in post tubercular knee treated with antibiotics and secondary closure. Patients with Rheumatoid arthritis (RA) are prone to develop the prosthetic joint infection.^17^ But we did not encounter such complications in our RA patients.

Early dislocation rate in total hip arthroplasty varies.^6,18^ But it is more common in the posterolateral approach. We also use the posterolateral approach and encountered one dislocation that was managed with closed reduction and skin traction for 4 weeks.

Common peroneal nerve (CPN) palsy in post-TKR is relatively rare, estimating around 0.4% that too is more common in Rheumatoid and valgus knees.^19,20^ We had also one lady with Rheumatoid and valgus knee where we performed simultaneous bilateral total knee arthroplasty in a single setting but she suffered from CPN palsy postoperatively in the left knee that recovered completely after 3 months of physiotherapy.

With an increase in the incidence of total joint replacements, there is a corresponding increase in the prevalence of periprosthetic fractures.^21^ We had two perioperative periprosthetic fractures during THR, one patient with Ankylosing spondylitis who underwent uncemented THR and one elderly osteoporotic lady with the cemented femoral stem. Both were Vancouver type A that was managed with circlage wiring and nonweight-bearing for 6 weeks.

Mortality after total joint replacement is mostly due to comorbid illness and pneumonia.^22,23^ We had three deaths. One man with bilateral knee replaced died after 2 years of surgery due to uncontrolled diabetes and hypertension. Another lady with bilateral knee replacement died one and a half years after she was diagnosed with lung cancer 6 months before her mortality. The third lady also died after 2 years of her right knee replaced due to an advanced stage of Parkinsonism.

The limitation of our study is it is a retrospective study with fewer sample size. It is also a single-center study. The socioeconomic status of the patients could not be assessed which is one of the major factors to undergo this type of surgery. A multicentric study with a larger sample size is recommended to validate the findings of our study.

## CONCLUSIONS

Total joint replacement of hip and knee is the ultimate solution for end-stage arthritis of these joints. There is no individual significant predictor of complications in patients subjected to total joint replacement. Singlestage bilateral TKR can be considered safe in regards to complications. Orthopedic surgeons and patients should be aware of possible complications and take necessary preventive measures.
